# *KAlignedoscope*: An interactive visualization tool for aligned clustering results from population structure analyses

**DOI:** 10.47248/hpgg2606020006

**Published:** 2026-03-27

**Authors:** Avery Guo, Sohini Ramachandran, Xiran Liu

**Affiliations:** 1 Division of Applied Mathematics, Brown University, Providence, RI, 02912, USA; 2 Painting, Rhode Island School of Design, Providence, RI, 02903, USA; 3 Data Science Institute, Brown University, Providence, RI, 02912, USA; 4 Department of Ecology, Evolution, and Organismal Biology, Brown University, Providence, RI, 02912, USA

**Keywords:** population structure, clustering, clustering alignment, visualization, interactive

## Abstract

Visualization plays an important role in the interpretation of analyses applied to population-genetic data, particularly when multiple clustering results are generated from the same input data and aligned to provide a comprehensive view of inferred population structure. We present KAlignedoscope, a web-based tool for the interactive visualization and exploration of aligned clustering results. Built with D3.js, our tool enables fast, dynamic rendering and offers powerful interactive features such as reordering populations and clusters, sorting individuals, highlighting clusters, and customizing colors. The tool is compatible with outputs from clustering alignment methods Clumppling and Pong, and is easily extendable to others. KAlignedoscope supports and streamlines population structure analysis by enabling flexible navigation of complex patterns in the aligned clustering results.

## 1. Introduction

A central goal in population genetics is to infer ancestry and population structure from multilocus genotype data for a sample of individuals. Researchers typically apply model-based clustering (e.g., Structure [1] and Admixture [2]) to an N×L genotype matrix (where N denotes number of genotyped individuals and L denotes number of loci genotyped), choosing a value for the number of latent clusters to be inferred (known as K). The output from a run of these clustering algorithms is a Q matrix, where the row vector ${\vec{q}}_{i}$ represents individual i’s estimated membership coefficients in each of the K clusters, which quantify the proportion of their genome derived from each latent ancestry cluster. Graphical illustration of estimated membership coefficients plays an important role in understanding the admixture patterns in individuals that are inferred from their genetic data. The most common graphic to show ancestry memberships is the “structure plot,” where each individual’s inferred membership coefficients in the latent clusters are shown as a stacked barplot of colored segments, with each segment’s length corresponding to the value of a membership coefficient and each color denoting a cluster for that clustering run. Tools such as DISTRUCT [3] have been developed to facilitate these visualizations.

Clustering runs often produce substantially different solutions from run to run due to factors like algorithmic stochasticity, label permutation, multiple optima, and varying algorithm settings (e.g., number of clusters, K). To jointly analyze and interpret results from *multiple clustering runs* of population structure inference, several **clustering alignment** methods have been developed [4–7]. Clustering alignment methods typically work by grouping runs with the same K value into “modes”, which represent distinct clustering solutions (sometimes with varying levels of support) for the genetic data [4–7]. For any given K, the mode to which most clustering runs align, i.e., the most frequent solution, is referred to as the “major mode”. Other “minor modes” often reveal alternative patterns that become clearer at higher values of K. Modes are then aligned across different K values to show how clusters emerge. Clumppling [7] supports systematic, pairwise alignment across K between all modes (major and minor) and reports alignment quality for these mode pairs. In contrast, earlier methods such as Clumpak [5] and Pong [6] align minor modes only to the major mode within the same K and align major modes across K; older tools such as Clumpp [4] are limited to aligning runs only with the same K value. The aligned modes are then typically visualized together in a series of structure plots arranged across increasing values of K. Previous methods, including Clumpak [5] and Pong [6], present these plots as a list, whereas Clumppling uses a graph-based layout with connecting edges to indicate the alignment quality (i.e., membership coefficient similarity) between modes across adjacent K values. By summarizing unique clustering solutions into modes and connecting modes with varying values of K, clustering alignment offers a more reliable and complete picture of inferred population structure.

Most clustering alignment methods display aligned modes as a vertical column of structure plots, with individuals aligned along the x-axis [4–6]. The most recent method, Clumppling [7,8], extends previous approaches by aligning all modes across different K, and presents the results as a multipartite graph of structure plots over a grid, where rows represent different K values and columns correspond to modes with the same K. While the new layout in Clumppling incorporates richer information, its Python-based rendering has limitations. The process can be slow (e.g., taking several minutes to generate a single figure for large datasets), and the resulting visualizations are static, offering limited flexibility in exploring different aspects of the results. In contrast, Pong [6] uses the JavaScript library Data-Driven Documents (D3.js) [9] to provide fast rendering with interactivity such as highlighting a single cluster of interest, albeit with a simplified column-style view that only displays major modes.

Clumppling and Pong produce similar output formats, motivating **a unified visualization tool** that can combine strengths of both methods into a single tool that is flexible to accommodate the output from any clustering alignment algorithm, supporting a much broader range of applications. We developed KAlignedoscope, an interactive visualization tool designed to display aligned clustering results in a fast, accessible, and user-friendly way, which facilitates interpretation of data and guides downstream analyses. With improvements in both user interface, customization options, and computational efficiency, this tool will benefit the population genetics community and any other field that utilizes clustering techniques.

## 2. Application

Our D3.js-based visualization tool KAlignedoscope has several key features, including interactively reordering individuals and clusters in structure plots, highlighting and displaying information for specific components, and customizing colors and text labels for displayed items. We summarized them in Table 1.

In addition to the main features listed in Table 1, KAlignedoscope was also designed to simplify live navigation and generate publication-quality figures. Users can navigate the structure plots and zoom in or out. The magnifier button resets the main plot, aligning the upper-left structure plot (i.e., the major mode with smallest K) with the canvas corner and restoring their default sizes so that all plots are in view. The full screen button allows users to expand the main canvas to the entire screen.

Upon downloading, KAlignedoscope produces two sets of figures. The primary download saves two PNG files, one for the cluster legend and one for the graph view of structure plots (excluding the user interface), as well as an SVG file for the graph view. Any custom change reflected in the user’s browser window will be maintained. Simultaneously, it opens a print dialog, allowing users to save a PDF of a snapshot of the entire webpage (including the user interface). The downloading process may take a few minutes, depending on both the size of the dataset and the web browser.

## 3. Implementation

Our tool is available as a light-weight Python package, KAlignedoscope, with minimal dependencies, easy installation, and straightforward usage. It is compatible with output from both Clumppling and Pong, and potentially with that of other clustering alignment methods. There are three inputs for the program: (1) the required input are files containing membership matrices of the aligned modes; (2) an optional file specifying the mode alignment quality (i.e., all pairs of modes with the alignment quality between them, similar to the one output by Clumppling) may be included to display alignment edges; (3) additionally, users may provide a labeling file containing the population label information for each individual, which will be used by KAlignedoscope to appropriately label the x-axes of the structure plots and enable the reordering and sorting functions for individuals.

**Figure 1.**
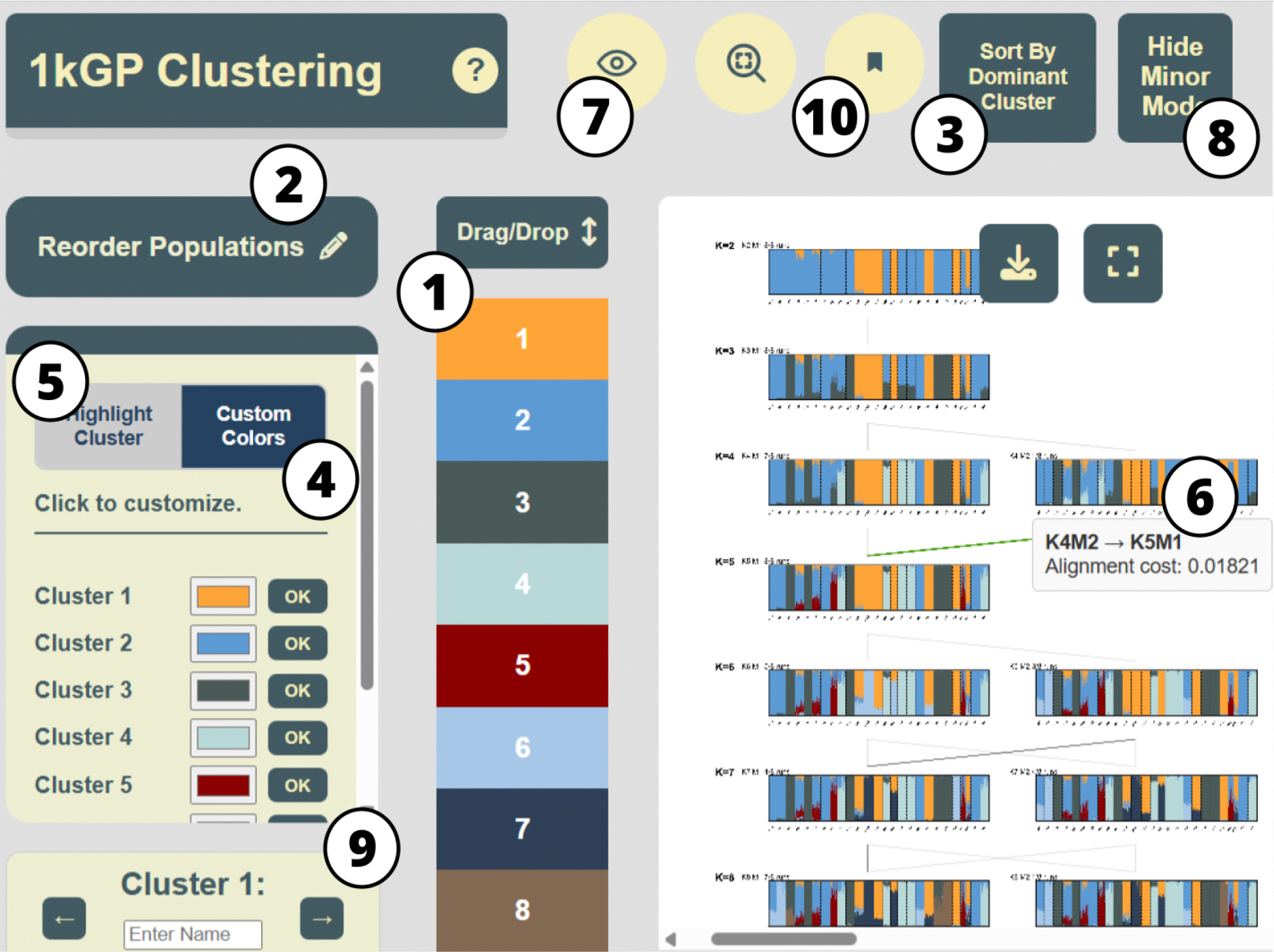
The KAlignedoscope user interface, with interactive features in Table 1 annotated by number. It displays the aligned clustering results produced by Clumppling on the 1000 Genomes Project data with 11 clustering modes from K=2 to 8. *Sort by Cluster Dominance* (Feature 3 in Table 1 ) was applied to the mode “K8M1” and its resulting individual ordering was propagated to all other modes. A tooltip for the alignment edge between modes “K4M2” and “K5M1” was activated, showing the corresponding alignment cost.

Figure 1 demonstrates the usage of our tool on aligned clustering results generated by Clumppling [7,8] (and Pong [6], available in the tool’s repository) on the Admixture [2] runs based on the 1000 Genomes Project data (Phase 3; [10]). This dataset was used in Behr et al. 2016 [6]; it includes 2,426 individuals from 26 populations. There are 8 clustering runs for each K from 2 to 8, resulting in a total of 56 runs. Figure 1 shows the user interface with the alignment results. The interactive features listed in Table 1 are annotated directly on the interface.

### 3.1. KAlignedoscope’s interface offers a comprehensive overview of aligned clustering results

Taking in the membership matrices of the aligned modes as well as the alignment patterns (both are output of the Clumppling program) as inputs, our tool presents the aligned modes in a connected graph of structure plots over a grid of number of clusters (K) and modes (M), similar to the visualization layout of Clumppling, in the main canvas of our user interface (Figure 1). Modes are labeled as “K3M1”, “K4M1”, etc., where for each K, the suffix M1 denotes the mode with the most clustering runs, M2 the second most, and so on. All interactive buttons and panels listed in Table 1 are annotated in this interface. Essential information is available upon opening the interface, and interactive features allow the exploration of more detailed information within each component.

### 3.2. Reordering and highlighting facilitate the examination of clustering patterns

**Figure 2.**
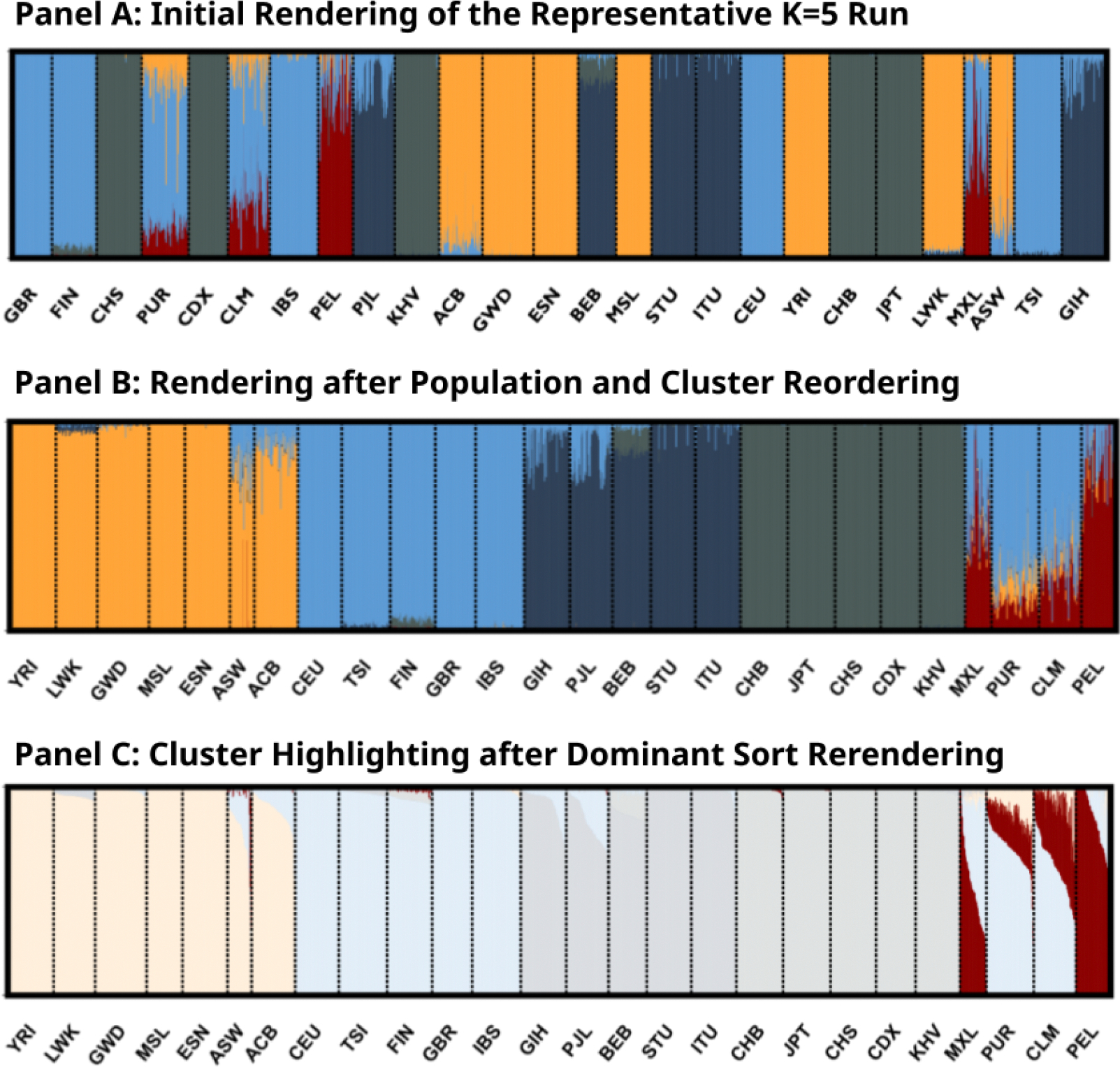
Zoomed-in structure plot of mode “K5M1” in Figure 1. (A) The default rendering of the clustering mode “K5M1” from Clumppling‘s output. (B) The same mode, with clusters reordered to place Cluster 2 (light blue) at the top and populations manually reordered to group similar cluster compositions (via *Reorder Clusters* and *Reorder Populations*, Features 1 and 2 in Table 1). (C) The same mode, with individuals sorted by their memberships in the largest cluster within each population, and Cluster 5 highlighted (via *Sort by Cluster Dominance* and *Highlight A Cluster*, Features 3 and 5 in Table 1).

We focus on the structure plot of “K5M1” (the major mode for K=5 runs) in Figure 2 to further demonstrate our interactive features. The initial rendering shown in Figure 2A uses the default ordering of individuals from the data. As a result, the clusters appear mixed and scattered, making it difficult to see the visual connections between them. KAlignedoscope addresses this issue with the option to *Reorder Populations* (Feature 2 in Table 1), illustrated in Figure 2B, which allows manually placing a population next to others that have similar cluster compositions and makes the visual patterns more recognizable.

*Highlighting A Cluster* (Feature 5 in Table 1) helps focus on a specific aspect of the results. For example, in Figure 2C, a single highlighted cluster is clearly most common among several Admixed American populations (PUR, MXL, PEL, and CLM) from the 1kG data, suggesting both shared admixture proportion and variation in this shared proportion among these groups. *Sorting by Cluster Dominance* (Feature 3 in Table 1) rearranges the individuals into descending orders of each population’s largest cluster, which creates a cleaner and more intuitive view of how clusters are distributed. For example, the highlighted cluster in Figure 2C is a minor component for populations PUR (Puerto Ricans in Puerto Rico) and CLM (Colombians from Medellín, Colombia), but is dominating the population PEL (Peruvians from Lima). Each interactive feature is useful for examining different aspects of the data. We encourage users to explore and experiment to find the visual options that best fit their specific needs.

## 4. Discussion

We developed KAlignedoscope, a tool for fast, interactive visualization of aligned clustering results from population genetic data. Our tool enables easy navigation through different aspects of clustering patterns and offers various options for customizing plot aesthetics. JavaScript D3 enables fast rendering of plots in the web, allowing users to quickly obtain clear and publication-ready visualizations of aligned clustering results. We designed our tool to be compatible with outputs from Clumppling and Pong, and easily extendable to other clustering alignment methods, making it broadly applicable to future methodological developments.

Visualization is not only a communication tool but also key for interpreting and evaluating population structure. Paired with population structure inference and clustering alignment methods, our tool streamlines population structure analysis workflows and provides comprehensive visualizations for exploring genetic admixture patterns.

## Figures and Tables

**Table 1.** Interactive features in KAlignedoscope.

	Feature	Description
1	Reorder Clusters *(drag and drop)*	A vertical legend on the left shows clusters in their current stacking order. The first cluster appears at the bottom of the structure plot, the second above it, and so on. Users can drag-and-drop cluster color blocks to reset a top-down order, which updates all plots with a quick re-render.
2	Reorder Populations *(drag and drop)*	Clicking the “Reorder Population” button gives a drop-down list of populations in their current ordering. This features is disabled if no population labels are provided. Users can move and reorder populations in the list. The updated order will be reflected in the x-axes of structure plots upon clicking the “Apply” button.
3	Sort by Cluster Dominance *(button click and selection)*	Clicking the “Sort by Cluster Dominance” button activates sorting of individuals based on a user-selected structure plot (i.e., a clustering mode) from the layout. Individuals within each population (if labels are provided) of the selected mode will be sorted by descending memberships in the cluster with largest total membership in that population. The same ordering of individuals will be applied to all other modes.
4	Customize Colors *(color picker)*	Users can assign new colors to clusters and apply them by clicking “OK” under the “Custom Colors” panel. By default, colors are drawn from a color-blind-friendly palette.
5	Highlight A Cluster *(mouse-over or click)*	Mousing over or clicking a cluster in the “Highlight Cluster” panel highlights it across all structure plots, while dimming the others.
6	Display Tooltip *(mouse-over)*	A tooltip (a small pop-up box) appears when hovering over individuals in a structure plot or over edges connecting aligned modes, displaying details such as an individual’s membership proportions or the alignment cost.
7	Show Alignment Edges *(button click)*	Clicking the eye button hides or shows edge connections between modes representing alignment costs, with darker edges indicating better alignment.
8	Hide Minor Modes *(button click)*	The default visualization shows all aligned modes. By clicking the “Hide Minor Mode” button, users can hide minor modes and show only major modes.
9	Rename Clusters *(toggle and in-line edit)*	In the bottom-left panel, users can rename clusters; changes will be synced to tooltips on the legend and to individuals’ bars across all structure plots.
10	Show Mode Labels *(button click)*	Clicking the bookmark button hides or shows the label of each mode, which includes the number of clusters, the mode name, and the number of clustering runs in the mode.

## Data Availability

Data presented in the study are included in the GitHub repository of the tool at https://github.com/ramachandran-lab/KAlignedoscope.
